# Determinants of Trust: Evidence from Elementary School Classrooms

**DOI:** 10.3390/jintelligence13120165

**Published:** 2025-12-15

**Authors:** Roberto Araya, Pablo González-Vicente

**Affiliations:** Institute of Education, CIAE, University of Chile, Santiago 8320000, Chile; pgonzalezvicente1@gmail.com

**Keywords:** trust, emotion, social-emotional skills, social cognition, personality traits, gender differences, education

## Abstract

Emotional intelligence (EI), specifically the capacity to recognize and understand one’s own emotions and those of others, is pivotal for developing the interpersonal skills that foster effective collaboration. This is especially crucial for developing trust in others, which serves as the necessary foundation for functioning in our increasingly impersonal contemporary society. Although extensive research has been conducted on trust in adults, empirical evidence for children remains limited. Quantifying the extent to which trust exists in young children, whether it differs from trust in adults, and how it changes with age, gender, and various psychological and school culture factors is essential for understanding how educational environments can foster its development. In this article, we analyze trust among almost 3000 fourth-grade children from 135 schools, measured based on behaviors exhibited during a Public Goods Game. The results align with other studies, showing that trust is substantially higher towards the in-group (classmates) than the out-group. A notable gender effect was observed, with boys exhibiting significantly higher levels of trust than girls. Trust was also higher in municipal schools compared to state-subsidized private schools. Personality traits, measured via the Big Five model using the Pictorial Personality Traits Questionnaire for Children (PPTQ-C), also emerged as influential. Specifically, elevated levels of Agreeableness and Conscientiousness predicted increased trust in both in-groups and out-groups. Extraversion and Openness to Experience also played a role, although to a lesser extent.

## 1. Introduction

In this study, we focus on one component of emotional intelligence, namely the social skills related to empathy and the understanding of others’ emotions. Emotional intelligence (EI) was initially conceptualized as a subset of social intelligences—the intra- and interpersonal intelligences from Howard Gardner’s Theory of Multiple Intelligences (Salovey and Mayer 1990, p. 189). It involves the ability to monitor one’s own emotions, to discriminate among them, and to use that information to support and guide thinking. This definition has been refined and operationalized through the Mayer–Salovey–Caruso Emotional Intelligence Test (MSCEIT and MSCEIT-2) (Mayer et al. 2025), which also includes the ability to regulate emotions in ways that benefit others. We focus on trust because mounting evidence indicates that emotional and social factors jointly influence children’s academic and life outcomes (Turan Bora and Akbaba Altun 2025). Given the important role that interpersonal skills play, the OECD incorporates these skills within the five domains of its Assessment Framework of Social and Emotional Skills for Better Lives (OECD 2018, 2024). One of these five domains is collaboration, which comprises the subdomains of empathy and trust. In individualistic societies, such as those in the West, trust is central to well-being, whereas in collectivist societies, empathy is more prevalent (Niu et al. 2023). Furthermore, trust in strangers is particularly critical for the emotional quality of interactions with unknown individuals (West et al. 2025) and for economic development and innovation (Henrich 2020). According to the OECD (2019, 2024), social-emotional skills are increasingly important for communities and nations as a whole, as they have been linked to higher levels of civic engagement, volunteering, and social integration, greater interpersonal trust and tolerance, and a decrease in antisocial and criminal behavior. Trust is a critical emotional skill. It is much more than an aspect of behavior and expectations; it is an emotional state that links representations of oneself, others, the situation, and emotion in a specific pattern of neural activation (Thagard 2018).

Trust is a complex concept comprising several dimensions. Hawley (2014) distinguishes between two principal aspects of trust. The first aspect, reliability, refers to the technical competence of people, artifacts, or institutional mechanisms as a “savoir faire” that provides assurance that things function as intended. The second aspect is interpersonal in nature, requiring transparency and honesty; violations of this aspect of trust typically have deeper and more long-lasting consequences. Interpersonal trust serves as the essential “glue” enabling institutions—conceived as collective minds—to operate effectively (Gallagher and Petracca 2024). In this study, we focus on interpersonal trust. Although early research employed vague conceptualizations of trust in others, subsequent scholarship has refined its constructs and methods of measurement.

### 1.1. The Two Types of Trust

The study of trust in others, both within in-groups and out-groups, has received considerable attention in recent decades (Savitskiy et al. 2025; National Academies 2023). Kumar et al. (2020) argue that understanding prosocial behavior is one of the greatest challenges of the twenty-first century. For example, Nguyen (2025) found that a one-standard-deviation increase in a CEO’s generalized trust is associated with 6% more future patents and a 4–6% increase in average patent quality. There is broad consensus among scholars regarding the necessity of distinguishing between these two forms of trust. Trust in relatives, friends, and acquaintances (in-group or close group) is generally greater than trust in strangers (out-group), particularly if one’s identity differs from that of the group (Delhey and Welzel 2012). Group size is critical for sustaining trust. As Burgess and Dunbar (2025) note, trust is a cognitive–economic process shaped by the memory cost of tracking past behavior and the frequency of policing intent. These constraints give rise to the fractal structure of human communities, making in-group trust fundamentally distinct from out-group trust.

Neuroscientific evidence suggests that trusting out-group members activates brain regions associated with top–down control, whereas trusting in-group members engages areas linked to reward processing (Hughes et al. 2017). This implies that trust in out-groups requires distinct cognitive and emotional mechanisms.

Explanations grounded in evolutionary biology (Hamilton 1964) and game theory (Axelrod 1984; Kumar et al. 2020) posit that trust within close groups evolved due to the need for cooperation among individuals who interact repeatedly, thereby discouraging deception.

However, trust in unknown third parties is the subject of wide debate (Chowdhury et al. 2025). One theory holds that trust in a close group is reinforced by increasing distrust in the outgroup and vice versa, such that both types of trust are opposed to a certain extent (Granovetter 1973; Stolle 1998). The association between them is thus negative.

Another theory posits that both types of trust share the same underlying substrate. According to this view, individuals exhibit a general propensity to trust, whether this tendency is innate (Becker 1996; Jones 1996) or acquired through socialization (Glanville and Paxton 2007). Because trust in unknown third parties is a simple extension of trust in the close group, those who are more trusting will also tend to be trusting of both the in-group and the out-group, and people who are less trusting will tend to show less trust in both groups. There is therefore a significant positive correlation between both types of trust.

Finally, there are those who maintain that trust in the out-group is not simply a projection of trust in the in-group but requires its own mechanisms, including economic, social, and cultural conditions (Delhey and Welzel 2012). According to this theory, there is a moderately positive correlation between the two types of trust, which varies culturally. What are these additional conditions that would enable the development of trust in the out-group? The rise of modernity may be one factor, as it requires trust in institutions, in the application of impartial norms, and in economic exchange in impersonal markets (Henrich 2020; Henderson 2024). Henrich et al. (2005) conducted behavioral experiments in 15 small-scale societies to test economic decision making across cultures. They found that trust and fairness in strangers varied widely, challenging the assumption of a universal “economic man”. Cooperative behavior correlated with market integration and community size, as societies more engaged in markets exhibited greater trust in anonymous others.

There have been several attempts to explain the nexus between the modernization of the family and the development of trust in the out-group (Kravtsova et al. 2024) or the development of democratic institutions and trust in the out-group (Siedler and Rainer 2006). Interestingly, Francois et al. (2018) report that increased intergroup competition can enhance both in-group and out-group trust. Their experimental data, along with analyses from the U.S. and Germany, indicate that individuals in more competitive environments, such as deregulated banking states or competitive industries, exhibit higher levels of trust. Francois et al. (2018) conducted a Public Goods Game that showed how levels of trust in the in-group increase when faced with increase intergroup competitiveness. Additionally, when asked at the end of the game if “most people can be trusted”, 68% of individuals faced with intergroup competition declared that they agreed, a much higher percentage than among those who did not compete (53%). The authors also analyzed data from the U.S. and Germany and found that as competition increases, in-group trust grows, but there is also an increase in out-group trust. This is the case with (i) employees who work in companies that face more competition, (ii) people who live in states where competition increases due to banking deregulation, and (iii) workers moving to more competitive industries (Francois et al. 2018).

### 1.2. The Measurement of Trust

The study of trust has a long history. Systematic quantitative research on trust dates back to the middle of the last century. The metric used by most surveys originates from a question devised by Noelle-Neumann in 1948, which measures trust using a single question: “Generally speaking, would you say that most people can be trusted, or that you should be very careful when dealing with people?” (Uslaner 2012). This approach has been criticized because it does not specify what is meant by “most people”. Interpretations can vary widely from one culture to another. Fukuyama (1999) proposed distinguishing between the radius of trust and the level of trust. “Radius of trust” refers to the width of the circle of people a respondent imagines as “the majority of people” (in most circumstances).

Building on this idea, Delhey et al. (2011) estimated the radius of trust in 51 countries, using the fifth round of the World Values Survey. This iteration of the survey incorporated, for the first time, six additional items measuring trust towards distinct social groups—three in-groups (relatives, neighbors, and personally known individuals) and three out-groups (strangers, individuals of different religions, and individuals of different nationalities). The researchers concluded that the radius of “most people” varied considerably from country to country; it was substantially narrower in Confucian countries and broader in high-income nations.

Other research on trust based on the World Values Survey determined that trust in the in-group is always positively associated with trust in the out-group, both individually and socially (Welzel 2010). However, despite their positive link, trust in the in-group and trust in the out-group represent two distinct dimensions of trust (Torpe and Lolle 2011).

In 2012, Delhey and Welzel (2012), using the World Values Survey, analyzed the relationships between trust in the in-group and trust in the out-group, making it clear that trust in the former is almost universal, while trust in the latter is highly variable between different countries and cultures. The results of the World Values Survey supported the third theory, showing a moderate positive correlation between the two types of trust but with considerable variation between countries. The authors also found that when society’s empowerment reduces in-group dependency, out-group trust surpasses the level projected by in-group trust (Delhey and Welzel 2012).

This second conclusion is supported by several other studies. Some emphasize the change in family structure, which, by reducing family size and diluting ironclad authoritarian control over its members, opens up new opportunities for individual empowerment and increased openness to the out-group (Kravtsova et al. 2024). Other research has emphasized the role of impersonal markets and democratic institutions as factors that stimulate trust in unknown third parties (Siedler and Rainer 2006).

### 1.3. Research on Trust in Children

Human social interaction is strongly influenced by a preference to consider the needs and interests of others. However, limited empirical evidence exists regarding the developmental trajectory of such preferences in childhood (Fehr et al. 2008). Most studies have been conducted on young people and adults, while the few studies on children tend to use small sample sizes (Sutter and Kocher 2007). Studying trust in children is crucial to deepening our understanding of the influence of cultural patterns on the development of these behaviors in adulthood. For this reason, we believe that this study, conducted with fourth-grade students (mean age: 10.2 years), makes an interesting contribution to the understanding of trust and its determining factors in children.

Our study included nearly 3000 students from 135 schools in the Metropolitan Region of Chile. This large sample of students spread over a large number of schools allows for a good representation of the children’s universe (at the age studied). Sample size is important, as it has been observed that small samples have limited power to establish firm results and can be easily biased (Bleidorn et al. 2022).

In our research, we theorized that two distinct types of trust exist: in-group trust and out-group trust. To measure them, we did not use the four-step intensity scale proposed in the World Values Survey; instead, we were inspired by experimental economics work (National Academies 2023) and used Public Goods Games, as explained in the next section.

There is also strong evidence that trait-based personality types predict outcomes in both the social and cognitive domains (Asendorpf and van Aken 1999; Thielmann et al. 2020). Therefore, we also incorporated that approach into this study.

In this paper, we examined trust in classmates (in-group) and in unknown students from other schools (out-group), quantifying the degree of trust in others using the Public Goods Game method. In the same sample, personality traits were measured using the Big Five model, allowing us to investigate the influence of these traits on the two types of trust.

- RQ1a: What level of trust exists in the in-group and in the out-group in fourth-grade students?
- RQ1b: What is the correlation between these two types of trust?
- RQ2: What sociobiological factors (gender, age, socioeconomic level, special teaching needs, type of school) influence levels of trust in fourth-grade students?
- RQ3: Do personality traits influence fourth-grade students’ level of trust? If so, how and to what extent?

## 2. Materials and Methods

### 2.1. Methodological Approach

This study’s methodological approach is grounded in experimental economics, which has undergone significant development in recent decades, building on the pioneering work of Smith in the 1960s and the contributions of the Grether–Plott and Kahneman–Tversky teams in the 1970s (Harrison 2002). Economic games have found wide application. Although these games are criticized for being imperfect predictors of real social behavior (Hui et al. 2024; Dorrough et al. 2020), their strength lies in the ability to isolate and test simple predictions (Benenson et al. 2007). Crucially, the information obtained from observed behavior is generally more reliable than information provided in questionnaires that directly ask about the degree of trust in others.

We used the Public Goods Game to measure both in-group and out-group trust. This methodology is widely used in the behavioral economics literature (National Academies 2023) and enables us to assess revealed behavior rather than relying solely on survey responses. In this study, we applied the Public Goods Game to children, a population for which empirical evidence on trust remains limited, enabling reliable and comparable measurement grounded in an established experimental framework.

The core methodology involved giving each student in the classroom 10 virtual flags and then asking each one how many of these flags they would donate to a common pool. Each child must decide how many flags to donate and how many to keep, with donation options ranging from none (*n* = 0) to all ten (*n* = 10) or any intermediate whole number. The student is informed that all flags accumulated in the common pool will be duplicated and then distributed equally among all students in the classroom.

As children place their flags into a common pool that is subsequently doubled, gains depend on mutual trust. The more flags each student contributes, the greater the collective benefit, provided mutual cooperation occurs. This decision requires trust, because if a student does not believe classmates will contribute to the common pool, the incentive is to free-ride by keeping most or all of the flags. An individual maximizes profit when others donate all of their flags but the individual donates nothing. Conversely, the worst situation occurs when the student donates everything and no one else does. This last possibility fosters distrust in others. A greater contribution indicates higher trust that others will also donate and will not behave selfishly. Thus, the number of flags donated serves as a behavioral proxy for trust. The game is then repeated to measure out-group trust, but, this time, the common pool is formed with students from a classroom in another school, who are unknown to the respondents. This dual design enables direct comparison between in-group and out-group trust levels.

The primary comparison criterion is the mean number of flags donated to the common pool in each case. More flags donated means greater trust. Additional analyses include the distribution of donations and the proportion of students who contributed zero flags, indicating complete distrust.

The virtual flags are highly valued by students of this age. Within mathematics lab classes, schools use the Conecta Ideas online platform (Araya and Diaz 2020; Ulloa and Araya 2020), where students receive virtual flags for two reasons: correct answers and helping peers (Araya and Gormaz 2021). For a correct answer on the first try, the student receives 3 flags. For a correct answer on the second try, they receive 2 flags, and if the answer is correct on the third try, they receive 1 flag. On the other hand, if a classmate asks for help, the student who provides help receives 4 flags if the help-seeker rates the help as satisfactory. Children frequently compare the number of accumulated flags.

Based on the number of flags accumulated over the month, students receive a hardcover book as a gift. The incentive structure has three conditions: simple, individual, and group. In the simple and individual conditions, the Conecta Ideas screen displays the accumulated flags for each student during the month, as well as the average accumulated flags per student in the class. In the group incentive condition, the Conecta Ideas screen displays the number of flags per capita for the class, along with the number of flags per capita for classes from other schools. In the individual condition, at the end of each month, a book is randomly awarded to 20% of the students in each classroom, with the probability proportional to each student’s accumulated number of flags. In the group incentive condition, 20% of the classes are randomly awarded, with probability proportional to the per capita rate, and all students from these classes receive a book. The use of flags makes this research viable without incurring large expenses.

### 2.2. Questionnaire and Method of Application

The Public Goods Game is administered by teachers in the classroom, under normal classroom conditions, using the Conecta Ideas platform, which they use weekly for mathematics. Classroom teachers and students are familiar with the platform as they use it weekly to answer both closed and open-ended questions. Every week, teachers review the exercises and questions beforehand, as well as any additional non-mathematical questions included on the platform that week. The advantage of this method is that a single teacher applies the test simultaneously to their entire class (with an average of 25 students per class) during the regular mathematics session. Consequently, no extra personnel were required to administer the Public Goods Game. Each student answers using a personal computer or tablet in the school lab using the Conecta Ideas platform, following the teacher’s instructions and ensuring the confidentiality of their responses. For survey questions, teachers read the question aloud, making sure that the entire class understands and answers the question presented. In this way, all students answer the questions individually without the teacher or other classmates knowing what they answered. Due to online connectivity failures, there are instances where questions must be administered on paper. In those cases, the answers were subsequently typed out by the teachers and added to the same database (11 schools out of 135).

Both Public Goods Game questions were applied identically; only the text of the question varied to reflect the change in group composition. This consistency strengthens the reliability of the comparison between in-group and out-group trust. Additionally, a benefit of this method is its efficiency and cost-effectiveness, which enables the use of large, geographically diverse samples. While this approach yields significant representational power, we acknowledge that the data’s quality may not be equivalent to that achieved in smaller studies where a dedicated psychologist or teacher administers the test one-on-one.

Below, we transcribe the text of each question.

**In-group trust question: Common pool within the class**.

We give you 10 little flags. You can donate to the common pool of your class. The common pool is doubled and distributed among all those in your class. How many little flags do you donate to the common pool?

**Out-group trust question: Common pool with a class from another school**.

We give you 10 little flags. You can donate to the common pool with a class from another school. The common pool is doubled and divided between all those in your class and the class of the other school. How many little flags do you donate to the common pool?

The teacher explains that the other class will do the same.

### 2.3. Sample

This study examined data collected from 4th-grade students from 135 public or state-subsidized private schools (often referred to as charter schools) in the Metropolitan Region (MR). This type of school enrolls 91% of the country’s students. No paid private schools were included in the sample. In our study, 52% of students attended a state-subsidized private school, and 48% attended a public school (managed by the municipality or a local public education service). These percentages closely mirror the national distribution.

School selection was not strictly probabilistic, as the Conecta Ideas platform is not available in all schools in the MR. Despite this limitation, the sample is highly representative of the socioeconomic reality of 91% of the country’s students, excluding the 9% who study in paid private schools and who, for the most part, belong to the highest income bracket. This was a large cross-sectional study conducted among students from medium–high, medium–low, and low socioeconomic backgrounds, with a percentage of vulnerability comparable to that of the MR population. Table 1 presents the vulnerability index of the sample compared to the MR population. This official index is only available for public and state-subsidized private schools, as it is not tracked for paid private institutions.

Data collection was managed by the participating schools. In total, 120 schools collected data between 18 and 24 November 2022, and 16 schools completed the study between 29 November and 7 December. Only one school experienced a delay, collecting data on 19 December 2022. One 4th-grade class was randomly sampled from each school. Thus, the total number of classes was 135.

For the first Public Goods Game question, 2924 students provided valid responses, and 2864 did so for the second. For the purpose of comparative analysis, we restricted the sample to the 2832 students who answered both questions validly. This restriction ensures that any observed statistical differences are solely attributable to differences in trust levels, rather than differences in sample composition (e.g., the distribution of different student profiles in each sample).

The resulting sample size (almost 3000 students) and broad representation (135 schools) are notable strengths, contrasting favorably with many smaller, less diverse studies on childhood behavior.

### 2.4. Profile of the Students in the Sample

The sampled students’ profiles are shown in Table 2. Age distribution was based on the age reached on the date of application of the Public Goods Game. The average age was 10.2 years.

Socioeconomic status (SES) was calculated by integrating two components. The first is the school-level vulnerability index (VI), the official Chilean government measure for school vulnerability, which includes socioeconomic factors and risk of school failure. The VI indicates the proportion of students in a situation of greater vulnerability within each school. The second component, an individual SES index, was computed using Principal Component Analysis (PCA) based on the following student-level variables: possession of certain goods (Internet, personal computer, or tablet), degree of overcrowding in the home, mother’s educational attainment, and the student’s educational aspirations.

These two indices were normalized and averaged to create a single composite SES index. Acknowledging that this is an imperfect, approximate measure, we created three broad SES categories: High (top 20% of scores), Medium (intermediate 60%), and Low (bottom 20%). This categorization was employed to compare trust levels between the two extreme groups of the distribution.

Almost 18% of students had Special Educational Needs (SEN), either temporary or permanent, and were enrolled in the School Integration Program (SIP) (see Table 2).

Schools were also classified according to religious orientation and administrative dependency (municipal or state-subsidized private). We also included student performance in mathematics, using the grade point average (GPA) for the year to date, which serves as a good proxy for overall school performance. In Chile, the grading scale ranges from 1 to 7, and students were categorized into three performance levels based on their GPA (see Table 3).

Finally, we utilized the personality data collected by the schools. These data were collected using the PPTQ-C (Pictorial Personality Traits Questionnaire for Children), as proposed by Maćkiewicz and Cieciuch (2016). The questionnaire is based on the Big Five personality trait model. The questionnaire was validated in a 2014 study with Polish students and has the advantage of being adapted to the cognitive level of schoolchildren, utilizing specific pictorial stimuli and 5-point response scales (Maćkiewicz and Cieciuch 2016). This adaptation is highly relevant, because personality traits are represented by images depicting behaviors familiar to students, thus avoiding abstract linguistic statements that could hinder comprehension. Furthermore, the character presented in each image was designed to be unisex, making the PPTQ-C suitable for use with both boys and girls. The Spanish version utilized by the schools was integrated into the Conecta Ideas online platform (Araya and González-Vicente 2023). This allowed students to be classified for each of the five Big Five personality traits, facilitating the analysis of their relationship with the predisposition to trust others. For each trait, students were categorized based on quartile distribution: 25% scoring High, 50% Medium, and 25% Low.

## 3. Results

**Figure 1:**
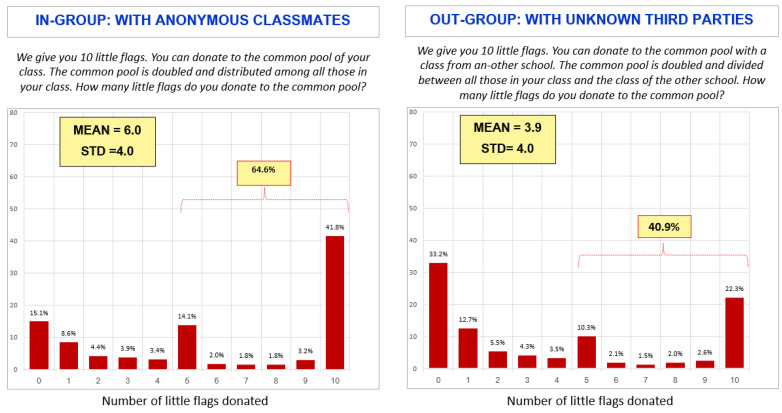
Distribution of the number of flags donated to the common pool.

Figure 1 illustrates the distribution of the number of flags donated to the common pool. The distribution is distinctly bimodal, with the highest frequencies concentrated at the extremes. This pattern, which is highly atypical of a normal distribution, suggests that students tend to be either highly trusting or highly distrusting.

### 3.1. Comparing Trust Types

The average number of flags given to the common pool is a useful indicator of trust for comparisons between groups (e.g., boys and girls). However, given the high variance of the distribution, we also include the percentage of students who donated five or more flags to the common pool.

The data revealed a clear distinction between in-group and out-group trust behaviors. We found that in-group trust (in classmates) was significantly higher than out-group trust (in unknown third parties from another school). This was an expected result and consistent with previous studies (see Table 4).

Conversely, in analyzing the level of distrust, only 15.1% of the students contributed zero flags to their class’s common pool. In other words, almost 85% showed at least a minimal degree of trust in their classmates. However, distrust in unknown third parties increased sharply, with 33.2% contributing zero flags to the out-group common pool. Thus, trust in the out-group fell from 85% to 66.8%.

### 3.2. Correlation Between Both Types of Trust

The correlation between both types of trust is an important indicator for understanding the potential relationship between them. The correlation was r = 0.42 (*p* < .001). This positive, intermediate value supports the hypothesis that trust in unknown third parties is neither a simple extension nor an antagonism of in-group trust. Instead, it suggests that trust in unknown third parties is a developed form of internal trust that requires additional factors or conditions to flourish. Table 5 shows the cross-tabulation between the two types of trust.

As shown in the table, 35.6% of the schoolchildren trusted both the in-group and the out-group, while 30.1% were rather distrustful towards both groups. The most interesting finding is the group that showed high trust towards the in-group but low trust towards strangers (29.0%). The inverse pattern (low ingroup, high out-group), on the other hand, rarely occurred (5.3%). These results show that trust in unknown third parties requires additional factors or conditions beyond trust in the in-group, as postulated.

### 3.3. Influence of Students’ Biosocial Traits on Trust

The influence of some biosocial traits on both types of trust was tested. We used ANOVA to compare the average number of flags given to the common pool in each of the two Public Goods Games. To determine the size of the effect of traits on trust, we used Cohen’s f^2^. A f^2^ ≥ 0.02 and lower than 0.15 is a small effect, f^2^ between 0.15 and 0.35 is a medium effect, and f^2^ ≥ 0.35 is a large effect. If the factor had more than two categories, we calculated the difference between the maximum and minimum values across all categories. The results are shown in Table 6.

We found a significant gender difference, as boys showed more trust than girls in both types of trust. This result is consistent with other studies, although there is no definitive consensus regarding the difference in trust between genders.

**Figure 2:**
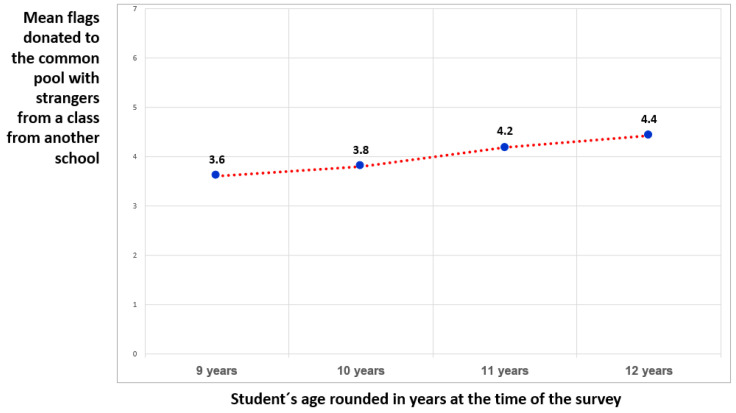
Out-group trust by age.

The results by age are interesting, as we did not detect differences in in-group trust; however, we did find differences in trust in unknown third parties, which increases with age. Figure 2 shows the detailed means by age, illustrating a slight but sustained increase in out-group trust as age increases.

We did not find differences according to socioeconomic level. However, it is worth noting that our study excluded private schools, which serve the population with the highest income and socioeconomic status. Therefore, the range of variation in socioeconomic status in our study is limited.

Students in the SIP, who have special education needs (whether temporary or permanent), exhibited a higher level of trust in others (both in the in-group and in the out-group) than non-SIP students. We cannot draw clear conclusions from this result, as most of the children in this program did not have a defined diagnosis. Among those who did, cases of specific language disorder (SLD), attention deficit hyperactivity disorder (ADHD), mild intellectual deficit (MID), and autism spectrum disorder (ASD) predominated. These are diverse diagnoses that do not allow for the extrapolation of results due to their great heterogeneity.

### 3.4. Influence of Personality Traits on Both Types of Trust

The “Big Five” model stipulates five major personality traits (McCrae and Costa 2009):

- Conscientiousness: Characterizes meticulous people with tenacity, self-discipline, and a desire to achieve.
- Agreeableness: Characterizes friendly, affable, and accommodating people, who avoid hostility and tend to get along well with others.
- Extroversion: Characterizes affectionate and loquacious people and lovers of fun; they are active and energetic.
- Neuroticism: Characterizes people who tend to experience negative emotions, such as worry and insecurity. Emotionally, they are labile rather than stable.
- Openness to Experience: Characterizes people who are curious, imaginative, intuitive, and original and who have broad interests.

Although the Big Five model is widely accepted and has been tested in young people and adults, research in children has been limited, due in part to the complexity of the questionnaires used. An important advance was the development of the PPTQ-C (Pictorial Personality Traits Questionnaire for Children), proposed by (Maćkiewicz and Cieciuch 2016). This questionnaire is based on pictorial scales, according to the level of cognitive development of children. In addition, the number of items considered for each personality trait has been considerably reduced, from 13 items in the adult questionnaire to only 3 items in the children’s questionnaire (Maćkiewicz and Cieciuch 2016).

Each personality trait is a latent variable calculated as a weighted average of the three items measured in the questionnaire. The weighting factor is determined by the normalized factor loads generated by the CFA model (in this case, the sum of the coefficients equals 1.0). For the purposes of our analysis, each latent variable (associated with each personality trait) was divided into three categories: High Level (25% of students who present the trait more strongly), Low Level (25% of students who present the trait more weakly), and Medium Level (the intermediate 50%).

Table 7 presents the levels of both types of trust in relation to personality traits. Agreeableness had the greatest impact on trust in the in-group. The 25% of students with the highest score in Agreeableness contributed an average of 6.6 flags to the common pool; at the other end, the 25% of students with the lowest score in Agreeableness contributed only 5.1 flags. This is a statistically significant and substantial difference, equivalent to 36% of the SD of in-group trust. Agreeableness had a similar effect on trust in unknown third parties (33% of the SD of out-group trust).

Furthermore, our research also shows an influence, albeit more moderate, of other personality traits on both types of trust: Conscientiousness, Openness to Experience (especially for in-group trust), and Extroversion (especially for out-group trust). Neuroticism (or its opposite, emotional stability) did not show a significant influence on either type of trust.

### 3.5. Influence of the School on Student Trust

We investigated the influence of the school on students’ trust in others using two variables: dependence on the school and their religious orientation.

In Chile, there are three types of schools, according to their financing: paid private schools (9% of school enrollment), usually high and upper-middle income; private schools that receive a subsidy from the state (54% of enrollment); and public schools, municipalities, or Local Education Services (37% of enrollment) (Centro de Prensa de Libertad y Desarrollo 2022). Our study encompassed the last two categories. Table 8 shows the results.

Students from municipal schools showed more trust than students from subsidized private schools, both in terms of trust in the in-group and in the out-group.

In Chile, municipal schools are secular; however, among state-subsidized private schools, there are both secular and religious institutions. Within the group of subsidized private schools, the mean level of trust among students did not differ from that of students attending religious schools.

### 3.6. Influence of School Performance on Both Types of Trust

Finally, we classified students into three groups according to their performance in mathematics, taking the average grade in that subject:

- High Performance: Students with an average math grade above 6.0.
- Medium Performance: Students with an average math grade between 5.01 and 6.0 (inclusive).
- Low Performance: Students with an average math grade equal to 5.0 or less.

We postulate that at this educational level, performance in mathematics is a good approximation of overall school performance. We investigated whether there was a relationship between students’ school performance and both types of trust, excluding students who, for various reasons, had not yet been graded in mathematics at the time of the test. No significant association was detected between the students’ school performance and their level of trust in others, either in the in-group or the out-group.

### 3.7. Revealed Trust and Declared Trust

The Conecta Ideas platform also has a sociometric module with several questions, including one about declared trust. Students were asked to indicate which classmates they trusted. They marked on the digital screen, from the list of classmates in their course, those they trusted. There were no restrictions; they could indicate all, some, or none of their classmates.

**Figure 3:**
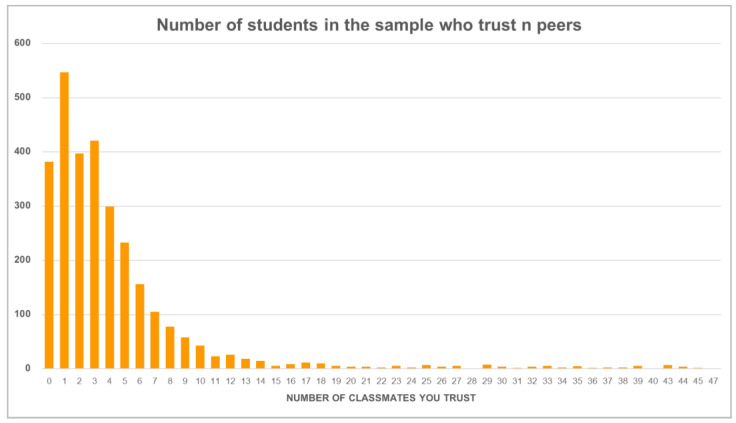
Distribution of the number of trusted classmates.

For each student, we counted the number of classmates trusted, which is a more personalized indicator of in-group trust. Figure 3 shows the distribution of the number of trusted classmates.

Most students only trusted a few classmates; the mean was 4.4, but the median was only 2.5, indicating the great asymmetry of the distribution. This large dispersion of responses also contributed to the high variance of the distribution (SD = 6.2). Because classes have different numbers of students, we normalized the number of trusted students by the size of the course, which transformed our indicator into a variable ranging from 0% to 100%.

This personalized trust indicator exhibited a positive correlation with general trust, measured by the Public Goods Game (r = 0.042, *p* < 0.05). Although the correlation was positive and statistically significant, it was very small. Our reasoning is as follows: if a student trusts a greater number of their classmates, they should be more willing to contribute a greater number of flags to the common pool, as this attitude expresses trust in the group. However, given the large dispersion of the data, which implies many values with a small number, this can only be appreciated by grouping the responses into broader and more robust categories. To test this hypothesis, we grouped students according to the percentage of peers they trusted and then calculated, for each group, the mean number of flags donated to the common pool.

**Figure 4:**
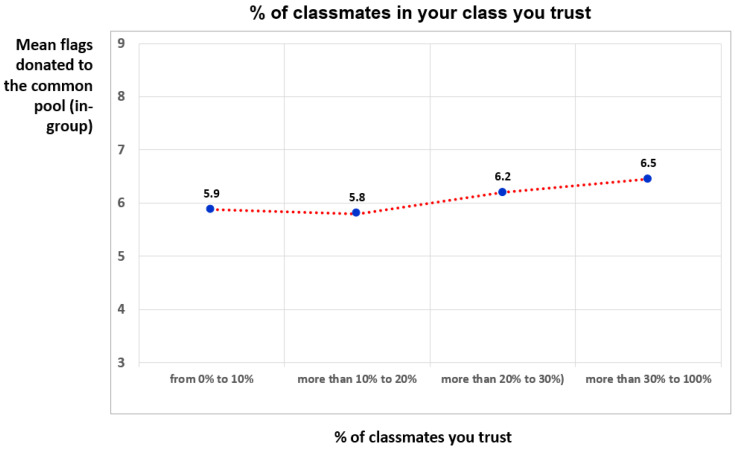
Average number of flags donated to the common pool according to the % of trusted classmates.

We recoded the data into four segments based on the number of classmates they trusted. We found that students who personally trusted more classmates donated more flags to the common pool. Figure 4 illustrates the results graphically, and Table 9 presents the results of the ANOVA test, which indicate that the mean difference between the four groups was statistically significant at *p* = 0.153 (F = 1.8). The results of the ANOVA test do not meet commonly accepted standards (*p* < .100 or *p* < .050). However, when the test was applied by grouping the first two segments, a statistically significant result was obtained (F = 2.5, *p* = .081). Likewise, the mean difference between the first two groups and the last two groups was statistically significant (t = 2.2, *p* = .03), as was the mean of group 4 compared to the first two groups.

## 4. Discussion

Reflecting the crucial importance of social and emotional skills, the OECD has recently proposed its Social and Emotional Skills Assessment Framework and begun measuring these skills in schools. This framework includes collaboration as one of its five core domains. Collaboration, in turn, includes the subdomains of empathy and trust (OECD 2018, 2024). While trust is fundamental to well-being in individualistic societies, empathy tends to prevail in collectivist contexts (Niu et al. 2023). Trust is a complex emotional state that integrates components of emotional intelligence as well as an individual’s perceptions of the emotional states of others. Furthermore, trust encompasses two distinct skills: neurological evidence demonstrates that the neural activation patterns associated with in-group trust are clearly different from those linked to out-group trust (Hughes et al. 2017). Despite their recognized importance for civic participation and social integration, large-scale international studies on these socio-emotional skills remain scarce.

Modern economic development depends on the extraordinary human ability to trust strangers across institutions, norms, and shared expectations. This impersonal trust enables large-scale cooperation and market exchange, forming the foundation of complex economic and social systems (Seabright 2004). Therefore, understanding how trust develops in childhood and how education can nurture is fundamental for both economic and democratic development. Schools are among the first institutions where children learn to cooperate beyond their families, interact with diverse peers, and build confidence in fairness and reciprocity. Other studies have investigated trust in children. For example, Harbaugh and Krause (2000) conducted classic Public Goods and Dictator Games with children aged six to twelve to investigate the development of altruism. In their version, children were divided into groups of four and given an initial endowment of tokens. In each round, they privately decided how many tokens to contribute to a common fund (the public good) and how many to keep for themselves (the private good). The experimenter then doubled the total contributions to the fund and divided the resulting amount equally among the four group members, independent of individual contributions. They found that children’s average contributions in the Public Goods Game were generally comparable to those of adults. However, to the best of our knowledge, our study is the first to study trust in children with a large sample size and to examine factors such as the differentiation of in-group trust from out-group trust, the type of school, and students’ personality profiles.

This study provides empirical evidence on the determinants of trust among children using a large and diverse sample of fourth-grade students in Chile. By employing a behavioral measure—the Public Goods Game—we were able to capture trust in action rather than relying solely on self-reported attitudes. This enhances the ecological validity of our findings.

### 4.1. Relationship Between In-Group and Out-Group Trust in Children

First, we addressed the research question RQ1a: What level of trust exists in the in-group and in the out-group in fourth-grade students? We confirmed a pattern previously established in the adult literature: trust in the in-group is significantly greater than trust in the out-group. This specifically translates into higher trust in classmates than in unknown third parties (represented by students from other schools). While this finding aligns with existing theory and was anticipated in this age group, this research makes a key contribution by quantifying the precise level of both in-group trust and out-group trust and measuring the quantifiable difference between these two forms of social trust among fourth-grade students. This quantification provides a crucial baseline for future studies exploring how this trust differential impacts behavioral and institutional outcomes in educational settings.

The seventh round of the World Values Survey (WVS), conducted between 2017 and 2022 among adults, revealed that trust in the in-group was consistently greater than trust in the out-group in all countries. In Chile, this survey was conducted at the beginning of 2018, and it showed that trust in neighbors was at 66% (the group closest to classmates, the in-group used in our study, where trust was 65%). Trust in unknown third parties was at 34% (in our study, trust in students from another school was 41%). The difference between the two types of trust in the WVS study in Chilean adults was 32 percentage points (Haerpfer et al. 2022). Our study, based on fourth-grade students using a different metric, showed a difference of 24 percentage points (Table 4). These differences depend on how each type of trust is measured and the metric used; therefore, the exact magnitude of the difference is less relevant than its order and direction. In both cases, the direction of the difference was the same: there was greater trust in the in-group than in the out-group, and the magnitude of the difference was substantial.

Regarding RQ1b, which addresses the correlation between these two types of trust, we found that in-group trust (trust in classmates) and out-group trust (trust in unknown students from other schools) are indeed correlated. Specifically, the correlation is positive and intermediate (r = 0.42) and statistically significant. This intermediate value is a key finding. It supports the theory that impersonal, out-group trust requires certain conditions and psychological mechanisms to develop, which are partially independent of those required for in-group trust. If both types of trust were simply dependent on the same general factors (e.g., general personality or parental behavior), we would expect the distance between them to be significantly smaller and the correlation to be much higher (closer to 1.0). The value of 0.42 suggests that while there is some shared psychological basis for believing others are trustworthy, trusting strangers occurs through a distinct developmental pathway.

What are the factors that allow for the development of trust in the out-group? This is a matter of debate, but we would expect that they are present among adults. We would also expect to observe significant variability in trust in the out-group across different countries. This is indeed the conclusion of several studies, including the World Values Survey. Furthermore, we would expect the difference between countries to be smaller with younger age groups. Unfortunately, there are no comparative studies in children large enough to confirm this conclusion. However, there are indirect indications that seem to support this idea. For example, a study in 12 countries on five continents in children aged 3 to 12 years showed that there were no differences in altruistic and prosocial behaviors between collectivist-oriented and individualist-oriented countries (Samek et al. 2020), contrary to what is observed in adult prosocial behavior (Delhey and Welzel 2012).

Prosocial behaviors tend to increase with age, as confirmed by numerous studies, which supports this idea. In the research mentioned earlier (Samek et al. 2020), for example, altruistic behavior, as measured by the Dictator Game, clearly increases with age among children aged 3 to 12 years. A similar phenomenon was observed in a study on altruism conducted among children aged 4, 6, and 9 in British schools using the Dictator Game (Benenson et al. 2007).

### 4.2. Trust in Children According to Their Age

Section 4.2, Section 4.3 and Section 4.4 address the second research question (RQ2): What sociobiological factors (gender, age, socioeconomic level, special teaching needs, type of school) influence fourth-grade students’ level of trust? These three sections describe how various student- and school-level demographics correlate with the measured levels of both in-group and out-group trust.

We found that trust in the in-group does not change significantly between the ages of 9 and 12. However, trust in the out-group grew slowly but systematically during the same age range. If this is corroborated in other countries, it would provide a strong argument in favor of the idea that trust in the out-group requires additional factors, as children absorb cultural norms that foster such an attitude. We cannot establish a firm conclusion, as our sample consisted of fourth-grade students who were not randomly sampled by age. As 11- and 12-year-old students are underrepresented at that school level, our sample may not be representative of this age group. Our findings largely align with those of other studies. For example, prosocial and altruistic behaviors grow with age from 3 to 12 years old (Samek et al. 2020), and trust increases almost linearly from early childhood to early adulthood (Sutter and Kocher 2007; Krabbendam et al. 2024). However, Kotaman and Aslan (2024) found no effect of age and gender on trust in kindergarteners.

### 4.3. Trust According to Children’s Gender

There is no consensus on the influence of gender on trust. Gilligan (1982) concluded that different genders approach moral problems differently; while women construct moral problems in terms of care and responsibility in relationships, men think of morality in terms of rights and rules. This would imply that women are more cooperative and committed to the community than men (Gilligan 1982). Later, Brown-Kruse and Hummels (1993) tested Gilligan’s idea, conducting a series of laboratory experiments to assess the effects of gender on trust and comparing their rates of contribution to the public good. They conducted the experiments on a group of undergraduate economics students and discovered, to their surprise, that it was men, not women, who contributed the most to the group’s common pool (Brown-Kruse and Hummels 1993). However, other researchers have obtained differing results. Cadsby and Maynes (1998) found no significant differences between men and women in the Public Goods Game (Cadsby and Maynes 1998) using a more diverse and extensive sample of university students. A possible explanation for these gender differences is the level of risk involved in these games (Dorrough et al. 2020). Nevertheless, these studies focused on adults.

We found significant differences by gender, as boys scored higher on both types of trust than girls, and this difference was more pronounced for in-group trust. This gender difference is particularly important for in-group trust (0.15 SD). One possible explanation is that boys frequently play games in which they act as a team against enemies (Benenson 2014). Even in the absence of explicit rivals, boys invent “enemies” during games. This is not observed in girls. This sense of competitiveness can make them more likely to collaborate with each other to compete with the external enemy. Following this line of thought, we could expect that boys develop greater in-group trust than girls, which is precisely what was observed in our research. This difference is consistent with another finding in the same sample. Using sociometric measurements (Mella-Defranchi and Araya 2025), we found a significant gender disparity in antipathy, as girls nominated an average of 5.27 disliked classmates, which was 1.45 more nominations than boys (effect size of 0.22 SD). This gender difference aligns with patterns of same-sex in-group aggression observed in team sports, where women are more likely to be excluded or receive aggression from teammates than men (Deaner et al. 2021).

The gender difference in out-group trust was also significant (0.10 SD). This difference could stem from two phenomena. First, as mentioned, boys frequently engage in combat team games against enemies, whereas girls do not (Benenson 2014). Second, there is abundant evidence that greater intergroup competition among voluntary associations, firms, and in experimental setups leads to higher trust in strangers (Henrich 2020; Francois et al. 2018). Combining these two facts suggests that boys’ greater exposure to team combat games against enemies may help explain their higher out-group trust compared to girls. However, further research on children is required to clarify the nature and robustness of these gender differences.

### 4.4. The Influence of the Type of School on Trust

We also found that students in municipal schools scored higher for both in-group and out-group trust than students in state-subsidized private schools. This indicates that there are general elements within schools that impact trust. It is possible that public education, due to a long tradition of teachers’ unions, places greater emphasis on values centered on solidarity and trust in others. It should be noted that some authors have found that not only membership in voluntary associations but also the type of association influences the degree of generalized trust observed in Public Goods Games (Degli Antoni and Grimalda 2014). However, other factors may also play a role, such as the type of family that chooses municipal education, the more diverse nature of municipal schools, a greater sense of school membership (Rinnooy Kan et al. 2023), or conditions perceived by individuals (Opp 1990). More research is needed to clarify these factors. In any case, to design public policies that promote prosocial behaviors in school children, this study reveals that certain school-related factors can make a significant difference by increasing confidence in classmates and strangers.

We also found greater trust in secular schools than religious schools, but this is qualified by the previous result, as municipal schools are secular. Therefore, if we restrict the analysis to subsidized private schools, where both secular schools and schools with a religious orientation (mainly Catholic) are present, the result is different: there is no significant difference between them with respect to trust in the in-group or the out-group. In both types of schools, trust was lower than in municipal schools. This result reinforces the idea that factors increasing trust in municipal schools could be related to the characteristics of municipal education. However, it is also concerning that in religious schools, where value formation is often outlined in their educational philosophy, this does not necessarily translate into a higher level of trust in others among students.

### 4.5. Trust and Personality Traits

Next, we address RQ3: Do the personality traits of fourth-grade students influence their trust in others? If so, how and to what extent? We found clear evidence confirming the influence of personal characteristics. While we previously noted that trust is influenced by sociocultural factors, such as gender and age, we found that students’ personality traits have a significant impact on both in-group and out-group trust. Specifically, we found that students ranking higher on the Agreeableness trait contributed more resources (flags) to the common pool. This result is not surprising, as there is substantial prior evidence that Agreeableness is a core trait that consistently promotes prosocial behaviors (OECD 2019), which include generosity and belief in the trustworthiness of others. This finding highlights the significance of personality in shaping early prosocial economic behaviors.

Our research also found a moderate influence of other personality traits on both types of trust, including Conscientiousness and Openness to Experience (especially for in-group trust) and Extroversion (especially for out-group trust). On the other hand, Neuroticism (or its opposite, emotional stability) did not significantly influence either type of trust. We can conclude that students with prosocial personality traits tend to be more trusting towards both in-groups and out-groups. This link between personality traits and levels of trust is of utmost importance, as people may be able to change their self-reported personality traits (Hudson and Fraley 2015). Thus, this link opens a way to strengthen trust in others. This could be an answer to the growing desire at the international level, particularly in OECD countries, for a holistic and more comprehensive educational policy that develops students’ cognitive and socioemotional skills (OECD 2018).

### 4.6. Trust Revealed and Declared

The Public Goods Game provides a general measure of trust in others. Although the metric has been specified and distinguishes between trust in acquaintances and trust in strangers, the Public Goods Game itself yields an abstract measure of trust, as the individual recipient is anonymous. What relationship should we expect between this measure of trust and a personalized trust indicator, where the anonymous subject acquires a face? Using sociometric data available on the Conecta Ideas platform, we correlated both measures of trust and found that the personalized trust indicator had a positive correlation with general trust, as measured by the Public Goods Game (r = 0.042, *p* < 0.05). It was a positive and statistically significant correlation, but of very small magnitude.

Although this result reflects the expected trend and correlation, it also highlights a distinction between the two measures of trust. Personal trust, defined here as trust in specific classmates by name, is more demanding and limited than the more abstract in-group trust, which incorporates elements of solidarity and altruism. This distinction may explain why even students who do not trust any classmates or only one of them are willing to donate flags to the common pool. This explanation is supported by our additional measurement of generosity (using the Dictator Game), where we found a significant correlation between in-group trust and generosity (r = 0.29, *p* < 0.001). In a future study, we will investigate the relationship between general trust in others and the level of personalized trust people exhibit towards their acquaintances.

## 5. Limitations and Future Work

This research is strengthened by an unusually large sample of students across numerous schools. Moreover, its field-based classroom measurements enhance ecological validity—and thus cognitive authenticity—by capturing naturalistic behaviors in the educational environment. In addition, it is easily repeatable in any school, starting from at least the fourth grade. This instrument could be very useful in determining and measuring the effects of various interventions that aim to improve trust among students, both within classes and within schools, and to improve classroom climate.

Among the study’s limitations, one is that it was conducted with the teacher present in the classroom, which may have introduced politeness and social desirability biases into the students’ responses. The presence of an adult in this type of research is unavoidable, given the students’ age. In some research, where an expert interviewer applies the survey, they withdraw for a moment or close their eyes as the child determines how many flags to donate. This method has advantages and disadvantages. In our case, although the teacher was present in the classroom, they did not monitor or control the students’ answers, as students answered through a digital medium from their designated positions within the classroom.

A more significant limitation is that the teacher explained the survey to the entire classroom, thereby precluding individual explanations for students who did not fully grasp the question or the mechanisms of the Public Goods Game. Although it is assumed that fourth-grade students can read and write, some of them experience difficulties with reading comprehension. This could imply that a part of the answers could lose validity if the instructions were not understood correctly. However, the fact that the results obtained did not differ significantly between students of high and low school performance, and that these results were quite consistent with the data from other research in children, suggest that this potential effect is unlikely to be substantial.

Measuring trust in strangers in educational settings is critical because it offers a quantifiable window into the development of prosocial skills—a foundational pillar for any thriving society. The distinction between trust in the in-group (classmates) and the out-group (unfamiliar students from other schools) profoundly conditions institutional outcomes.

When societies maintain a large trust gap (high in-group but low out-group trust), vital institutions, such as democracy, financial markets, and innovation, stagnate, as cooperation is restricted to narrow circles. Conversely, when trust extends broadly to strangers, this supports impersonal rules, universal values, large-scale cooperation, civic participation, institutional effectiveness, and innovation (Henrich 2020; Seabright 2004). By identifying and measuring this trust differential in children, we gain the knowledge necessary to design targeted educational interventions that actively foster generalized trust and enable collective social and economic prosperity. Our future work will continue this line of inquiry, extending the analysis to other prosocial skills, such as generosity and honesty. Our long-term aim is to help identify precise classroom strategies and educational mechanisms that can effectively nurture and develop these essential prosocial skills.

In conclusion, this study contributes to the growing body of literature on trust by offering novel evidence from a child population, employing a rigorous behavioral methodology, and highlighting the roles of personality and school context. Understanding how trust develops in early life is essential for building more cohesive and cooperative societies.

## Figures and Tables

**Table 1:** Vulnerability index (VI) of the sample and schools of the Metropolitan Region of Chile (RM).

**Vulnerability Index**	**Sample**	**MR**
Under 35	--	1.1%
From 35 to 44	1.9%	1.8%
From 45 to 54	3.3%	3.4%
From 55 to 65	3.2%	5.9%
From 65 to 74	18.0%	13.2%
From 75 to 84	28.8%	25.0%
From 85 to 94	41.6%	42.5%
From 95 to 100	3.2%	7.1%
Sum	100%	100%
AVERAGE VI	81%	80%

**Table 2:** Profile of the students surveyed.

Gender	Number of Students (n)	Relative Frequency (%)
Male	1.497	52.9%
Female	1.335	47.1%
Age (met)	N	%
9 years	64	2.2%
10 years	2.209	78.0%
11 years	478	16.9%
12 years	81	2.9%
Socioeconomic level	N	%
High level	595	21.0%
Intermediate level	1.672	59.0%
Low level	565	20.0%
Special Educational Needs	N	%
Yes (SIP)	499	17.6%
No	2.320	81.9%
Not classified	13	0.5%
Total	2.832	100%

**Table 3:** Profile of the schools surveyed and student performance.

School Dependency	Number of Students (n)	Relative Frequency (%)
Municipal (all types)	1.375	48.6%
Subsidized private	1.457	51.4%
Religious orientation	N	%
Lay schools	2.122	74.9%
With religious orientation	710	25.1%
Student performance	N	%
Low (grade ≤ 5)	608	21.5%
Middle (5 < grade ≤ 6)	960	33.9%
High (grade > 6)	858	30.3%
Unreported rating	406	14.3%
Total	2.832	100%

**Table 4:** Comparison of in-group and out-group trust levels.

Statistics	Trust in the In-Group	Trust in the Out-Group	Significant Difference	Significance Test
Mean flags donated	6.0	3.9	Yes (0.000)	T-test for dependent samples
% of students who donated 5 flags or more	64.6%	40.9%	Yes (0.000)	Comparison of percentages for the same sample

**Table 5:** Cross-tabulation of in-group and out-group Trust.

Trust in the Out-Group	Trust in the In-Group		
	Low (0–4 Flags)	High (5–10 Flags)	Total
Low (0–4 flags)	30.1%	29.0%	59.1%
High (5–10 flags)	5.3%	35.6%	40.9%
Total	35.4%	64.6%	100.0%

**Table 6:** Influence of students’ biosocial traits on trust levels.

Variable	Categories or Levels	In-Group Trust		Out-Group Trust	
		Mean Flags Donated	Effect Size	Mean Flags Donated	Effect Size
Gender	Male	6.3	0.15 (Sig = 0.00)	4.1	0.10 (Sig = 0.01)
	Female	5.7		3.7	
Age	9–10 years	6.0	0.02 (No Sig)	3.8	0.11 (Sig = 0.02)
	11 years or older	6.1		4.2	
Socioeconomic Level	High level	5.6	0.13 (No Sig)	3.9	0.03 (No Sig)
	Medium level	6.1		3.8	
	Low level	6.1		4.0	
Special Educational Needs (SEN)	Yes (SIP)	6.3	0.10 (Sig = 0.04)	4.5	0.19 (Sig = 0.00)
	No (SIP)	5.9		3.7	

**Table 7:** Influence of personality traits on both types of trust.

Variable	Categories or Levels	In-Group Trust		Out-Group Trust	
		Mean Flags Donated	Effect Size	Mean Flags Donated	Effect Size
Conscientiousness	High Level (25%)	6.6	0.27 (Sig = 0.00)	4.3	0.20 (Sig = 0.00)
	Medium Level (50%)	5.9		3.8	
	Low Level (25%)	5.5		3.5	
Agreeableness	High Level (25%)	6.6	0.36 (Sig = 0.00)	4.4	0.33 (Sig = 0.00)
	Medium Level (50%)	6.2		4.0	
	Low Level (25%)	5.1		3.0	
Extroversion	High Level (25%)	6.3	0.14 (Sig = 0.05)	4.3	0.19 (Sig = 0.00)
	Medium Level (50%)	6.0		3.8	
	Low Level (25%)	5.7		3.5	
Neuroticism	High Level (25%)	5.8	0.11 (Sig = 0.18)	3.7	0.11 (Sig = 0.12)
	Medium Level (50%)	6.0		3.8	
	Low Level (25%)	6.2		4.1	
Openness to Experience	High Level (25%)	6.3	0.17 (Sig = 0.01)	4.3	0.16 (Sig = 0.01)
	Medium Level (50%)	6.0		3.8	
	Low Level (25%)	5.6		3.6	

**Table 8:** Influence of the school on both types of trust.

Variable	Categories	In-Group Trust		Out-Group Trust	
		Mean Flags Donated	Effect Size	Mean Flags Donated	Effect Size
School Dependency	Municipal (all types)	6.4	0.19 (Sig = 0.00)	4.3	0.21 (sig = 0.00)
	Subsidized private	5.6		3.5	
Religious Orientation (Subsidized Private Schools)	Lay schools	5.6	0.02 (No sig)	3.5	0.03 (No sig)
	Religious orientation	5.5		3.4	

**Table 9:** Mean number of flags contributed to the common pool according to the percentage of classmates trusted.

Segments	Number of Students (N)	Mean Contributed to the Common Pool	Standard Deviation (SD)
From 0% to 10%	1.686	5.90	4.06
More than 10% to 20%	654	5.81	3.95
More than 20% to 30%	210	6.20	3.91
More than 30% up to 100%	220	6.45	3.96
Total	2.770	5.94	4.02

## Data Availability

The data presented in this study are available in in ConectaSTEM at https://conectastem.cl/data-fron-a-trust-study-3000-chilean/, accessed on 10 December 2025.

## Abbreviations

The following abbreviations are used in this manuscript:

MR	Metropolitan Region
SIP	School Integration Program
SEN	Special Educational Needs
VI	Vulnerability Index
